# Supplementary material for: Precise modulation of transcription factor levels identifies features underlying dosage sensitivity
Source: Nat Genet. 2023 Apr 6;55(5):841–51. doi: 10.1038/s41588-023-01366-2 (PMC10181932; doi:10.1038/s41588-023-01366-2)
Supplement: Supplementary file 1 — Supplementary Fig. 1. [file 41588_2023_1366_MOESM1_ESM.pdf]

nature genetics

Article

https://doi.org/10.1038/s41588-023-01366-2

Precise modulation of transcription factor levels identifies features underlying dosage sensitivity

In the format provided by the authors and unedited

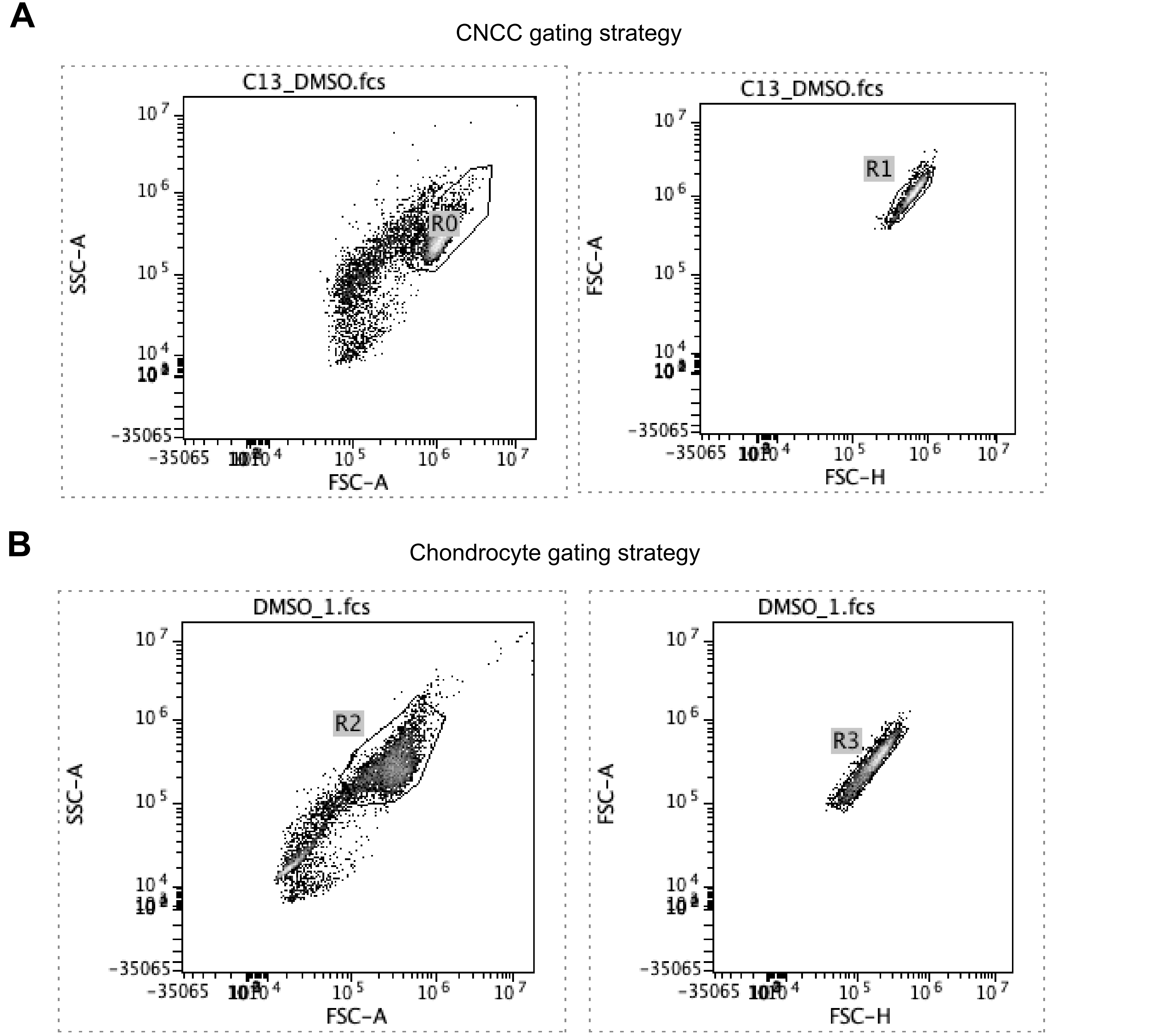

Supplmentary Figure 1. Example gating strategy for viable, single CNCCs (A) or chondrocytes (B) based on forward (FSC) and side (SSC) scatter area (A) and height (H)
