# Supplementary material for: Precise modulation of transcription factor levels identifies features underlying dosage sensitivity
Source: Nat Genet. 2023 Apr 6;55(5):841–51. doi: 10.1038/s41588-023-01366-2 (PMC10181932; doi:10.1038/s41588-023-01366-2)
Supplement: Supplementary file 2 — Reporting Summary [file 41588_2023_1366_MOESM2_ESM.pdf]

nature portfolio

Corresponding author(s): Jonathan Pritchard, Joanna Wysocka

Last updated by author(s): Feb 4, 2023

Reporting Summary

Nature Portfolio wishes to improve the reproducibility of the work that we publish. This form provides structure for consistency and transparency in reporting. For further information on Nature Portfolio policies, see our Editorial Policies and the Editorial Policy Checklist.

Statistics

For all statistical analyses, confirm that the following items are present in the figure legend, table legend, main text, or Methods section.

n/a	Confirmed	
☐	☒	The exact sample size (*n*) for each experimental group/condition, given as a discrete number and unit of measurement
☐	☒	A statement on whether measurements were taken from distinct samples or whether the same sample was measured repeatedly
☐	☒	The statistical test(s) used AND whether they are one- or two-sided<br>*Only common tests should be described solely by name; describe more complex techniques in the Methods section.*
☐	☒	A description of all covariates tested
☐	☒	A description of any assumptions or corrections, such as tests of normality and adjustment for multiple comparisons
☐	☒	A full description of the statistical parameters including central tendency (e.g. means) or other basic estimates (e.g. regression coefficient) AND variation (e.g. standard deviation) or associated estimates of uncertainty (e.g. confidence intervals)
☐	☒	For null hypothesis testing, the test statistic (e.g. *F*, *t*, *r*) with confidence intervals, effect sizes, degrees of freedom and *P* value noted<br>*Give P values as exact values whenever suitable.*
☒	☐	For Bayesian analysis, information on the choice of priors and Markov chain Monte Carlo settings
☒	☐	For hierarchical and complex designs, identification of the appropriate level for tests and full reporting of outcomes
☒	☐	Estimates of effect sizes (e.g. Cohen's *d*, Pearson's *r*), indicating how they were calculated

Our web collection on statistics for biologists contains articles on many of the points above.

Software and code

Policy information about availability of computer code

Data collection	Flow Cytometry Data Collection: CytExpert Acquisition and Analysis Software v2.4. Flow cytometry analysis: FCSalyzer v0.9.18
Data analysis	Custom code used for analysis of processed sequencing data is available on Zenodo9. KU Leuven provides the MeshMonk (v.0.0.6) spatially dense facial-mapping software, free to use for academic purposes (https://github.com/TheWebMonks/meshmonk)10. Matlab 2017b implementations of the hierarchical spectral clustering to obtain facial segmentations are available on Figshare 11.The following versions of software were used: skewer v0.2.2; bowtie2 v2.4.1; samtools v1.10; deeptools v3.5.0; MACS2 v2.2.7.1; fimo v5.1.1; slamdunk v0.4.3; salmon v1.4.0;

For manuscripts utilizing custom algorithms or software that are central to the research but not yet described in published literature, software must be made available to editors and reviewers. We strongly encourage code deposition in a community repository (e.g. GitHub). See the Nature Portfolio guidelines for submitting code & software for further information.

Data

Policy information about availability of data

All manuscripts must include a data availability statement. This statement should provide the following information, where applicable:

- Accession codes, unique identifiers, or web links for publicly available datasets
- A description of any restrictions on data availability
- For clinical datasets or third party data, please ensure that the statement adheres to our policy

The raw sequencing files generated during this study are available on GEO (accession number GSE205904); corresponding processed data is available on Zenodo9. Transcription factor binding motifs were obtained from HOCOMOCO v11 (https://hocomoco11.autosome.org/). Gene ontology assignments were obtained from AmiGO (http://amigo.geneontology.org/amigo). All analyses were done on human genome version hg38, except for PRS endophenotype GWAS (hg19). The raw source data for the facial phenotypes -the 3D facial surface models in.obj format- are available through the FaceBase Consortium (www.facebase.org). Access to these 3D facial surface models requires proper institutional ethics approval and approval from the FaceBase data access committee. Facial scans from PRS patients (used to define the PRS endophenotype) are available through the FaceBase Consortium (https://www.facebase.org FB00000861) under controlled access. The participants making up the US dataset of healthy individuals used for PRS endophenotype GWAS were not collected with broad data sharing consent. Given the highly identifiable nature of both facial and genomic information and unresolved issues regarding risks to participants of inherent reidentification, participants were not consented for inclusion in public repositories or the posting of individual data. This restriction is not because of any personal or commercial interests. Further information about access to the raw 3D facial images and/or genomic data can be obtained from the PSU IRB (IRB-ORP@psu.edu, and the IUPUI IRB (irb@iu.edu). The ALSPAC (UK) data will be made available to bona fide researchers on application to the ALSPAC Executive Committee (http://www.bris.ac.uk/alspac/researchers/dataaccess). Summary statistics from the PRS endophenotype GWAS are available on GWAS Catalog (GCP000517).

Human research participants

Policy information about studies involving human research participants and Sex and Gender in Research.

Reporting on sex and gender

Both males and females were included in both the control sample of 8,246 healthy individuals (60.3% female, remainder male) and the sample of Pierre Robin Sequence (9 female, 4 male). Sex was based on self-reporting

Population characteristics

The control sample of healthy individuals comprised three-dimensional facial scans of 8,246 unrelated individuals of European ancestry (60.3% female; median age = 18.0 years, IQR = 9.0 years) originating from the US and the UK. The sample of Pierre Robin Sequence comprised 13 participants (9 female; median age = 12.01 years, IQR = 5.17 years).

Recruitment

See White et al, Nature Genetics 2021 for details on recruitment of the control sample of 8,246 healthy individuals. Pierre Robin Sequence individuals were enrolled as part of a larger study following syndromic clinical and/or genetic diagnosis (see https://www.facebase.org/chaise/record/#1/isa:dataset/RID=TJ0)

Ethics oversight

Collection of data from PRS patients was carried out with overall approval and oversight of the Colorado Multiple Institutional Review Board (IRB #09–0731), was additionally approved by the institutional review boards of the University of Calgary, Florida State University, the University of California San Francisco, and the Catholic University of Health and Allied Sciences (Mwanza, Tanzania), and was carried out with the approval of the National Institute for Medical Research (Tanzania). Written informed consent was obtained from all study subjects or their parents, as appropriate. No subjects received compensation.

The PRS endophenotype GWAS in this study was conducted on individuals of European ancestry. The conclusions of this GWAS therefore may not be applicable to individuals of other, diverse ancestries. For the PRS endophenotype GWAS conducted in healthy individuals, ethical approval was obtained at each recruitment site and all participants gave their written informed consent prior to participation. For individuals under 18 years of age, written consent was obtained from a parent or legal guardian. For the US sample, the following local ethics approvals were obtained: Pittsburgh, PA (PITT IRB #PRO09060553 and #RB0405013); Seattle, WA (Seattle Children's IRB #12107); Houston, TX (UT Health Committee for the Protection of Human Subjects #HSC-DB-09-0508); Iowa City, IA (University of Iowa Human Subjects Office IRB #200912764 and #200710721); Urbana-Champaign, IL (PSU IRB #13103); New York, NY (PSU IRB #45727); Cincinnati, OH (UC IRB #2015–3073); Twinsburg, OH (PSU IRB #2503); State College, PA (PSU IRB #44929 and #4320); Austin, TX (PSU IRB #44929); San Antonio, TX (PSU IRB #1278); Indianapolis, IN and Twinsburg, OH (IUPUI IRB #1409306349). For the UK sample, ethical approval for the study (Project B2261: "Exploring distinctive facial features and their association with known candidate variants") was obtained from the ALSPAC Ethics and Law Committee and the Local Research Ethics Committees. Informed consent for the use of data collected via questionnaires and clinics was obtained from participants following the recommendations of the ALSPAC Ethics and Law Committee at the time. Consent for biological samples has been collected in accordance with the Human Tissue Act (2004).

Note that full information on the approval of the study protocol must also be provided in the manuscript.

Field-specific reporting

Please select the one below that is the best fit for your research. If you are not sure, read the appropriate sections before making your selection.

☒ Life sciences ☐ Behavioural & social sciences ☐ Ecological, evolutionary & environmental sciences

For a reference copy of the document with all sections, see nature.com/documents/nr-reporting-summary-flat.pdf

Life sciences study design

All studies must disclose on these points even when the disclosure is negative.

Sample size	Seven independent biological replicates were used for ATAC-seq and RNA-seq analysis in differing SOX9 concentrations, as a higher degree of accuracy was desired than typical in the field for modeling quantitative changes at individual regulatory elements/genes. Six chondrocyte differentiation replicates were used for sGAG assays, as similar accuracy for detecting quantitative changes was desired. Two independent biological replicates were used for 3h or 24h ATAC and H3K27ac depletion experiments, similar to other studies in the field, where large effects can be easily detected at this sample size. V5 and TWIST1 ChIP-seq were performed with two biological replicates, as the goal was to assess trends in signal across many regulatory elements as a group, rather than individually with high accuracy. Three independent biological replicates were used for 3h or 24h SLAM-seq full depletion experiments due to the lower sequencing depth (and thus likely lower statistical power) expected with nascent RNA sequencing. 2-4 independent biological replicates were used for flow cytometry-based analysis of SOX9 protein levels as a function of dTAG concentration.
Data exclusions	Two RNA-seq samples (WT_R8_5e-7M and S9CC47_R6_5e-7M) were identified as extreme outliers in initial principle component analysis. This was confirmed to be due to a library quality issue and so these samples were excluded prior to any further downstream analyses. When fitting Hill equation or linear models to each RE/gene, individual sample outliers with a z-score greater than 3 for that RE/gene only were removed.<br><br>For PRS endophenotype GWAS, images were excluded if participants were laughing, crying or otherwise emoting or judged to be of poor quality or if the non-rigid registration failed. Participants with missing covariate information (e.g. age, sex) were additionally removed
Replication	Western blots, DNA gels, and representative flow cytometry analyses were run twice, independently, with similar results.<br><br>To ensure reproducibility of the fitted ED50 values for each RE/gene, which form the basis of many analyses in the manuscript, a bootstrapping procedure was used which randomly resampled data points at each SOX9 concentration. This was used to construct 95% confidence intervals when comparing ED50 values between groups of genes. The strong enrichment for known SOX9 motifs in both SOX9-dependent and SOX9-bound REs also served as biological validation for those experiments. The presence of TWIST binding motifs from TWIST1 ChIP-seq data served as biological validation. The high correlation between effects of full SOX9 depletion (at 3h or 24h) with full SOX9 depletion at 28h on ATAC-seq signal served as independent validation. The fact that all SNPs specifically affecting the PRS endophenotype had been previously identified and replicated in other studies for different facial phenotypes served as independent validation. The high correlation between the effects of 24h SOX9 depletion of nascent transcription (SLAM-seq) and 48h depletion on mRNA (RNA-seq) served as independent validation.
Randomization	Samples were assigned to groups based on treatment conditions and cell line genotype. RNA-seq ATAC-seq and H3K27ac ChIP-seq counts were adjusted for differentiation batch effects using the DESeq2 framework.
Blinding	No blinding was done, but all samples were processed and analyzed equally

Reporting for specific materials, systems and methods

We require information from authors about some types of materials, experimental systems and methods used in many studies. Here, indicate whether each material, system or method listed is relevant to your study. If you are not sure if a list item applies to your research, read the appropriate section before selecting a response.

Materials & experimental systems

n/a	Involved in the study
☐	☒ Antibodies
☐	☒ Eukaryotic cell lines
☒	☐ Palaeontology and archaeology
☒	☐ Animals and other organisms
☒	☐ Clinical data
☒	☐ Dual use research of concern

Methods

n/a	Involved in the study
☐	☒ ChIP-seq
☐	☒ Flow cytometry
☒	☐ MRI-based neuroimaging

Antibodies

Antibodies used	Abcam rabbit anti-V5 (ab9116, ab15828), ActiveMotif anti-H3K27ac (39133), Abcam mouse anti-TWIST1 (ab50887), Sigma-Aldrich rabbit anti-SOX9 (AB5535); Abcam HRP anti-beta Actin (ab49900)
Validation	For rabbit anti-V5, ChIP-seq was performed in cells with present or depleted SOX9, and decreased signal was observed in the SOX9-depleted cells (see Extended Data Figure 4b). For rabbit anti-SOX9, Western blotting of protein lysates was performed in cells with present or depleted SOX9, and no signal was observed in the SOX9-depleted cells (see Figure 1c). See ActiveMotif website (https://www.activemotif.com/catalog/details/39133/histone-h3-acetyl-lys27-antibody-pab) for anti-H3K27ac ChIP-seq validation. See Abcam website (https://www.abcam.com/twist-antibody-twist2c1a-ab50887.html) for anti-TWIST1 ChIP-seq validation. See Abcam website (https://www.abcam.com/hrp-beta-actin-antibody-ac-15-ab49900.html) for anti-beta Actin Western blot validation.

March 2021

Eukaryotic cell lines

Policy information about cell lines and Sex and Gender in Research

Cell line source(s)	WA09 (H9) hESCs: Wicell, female. HEK293FT (R70007): Invitrogen, female.
Authentication	H9 hESCs and HEK293FT cells were obtained commercially and validated by their commercial source (WiCell for H9, Invitrogen for HEK293FT) by karyotyping, STR profiling, and marker expression
Mycoplasma contamination	All lines tested negative for mycoplasma contamination
Commonly misidentified lines (See ICLAC register)	None

ChIP-seq

Data deposition

☒ Confirm that both raw and final processed data have been deposited in a public database such as GEO.

☒ Confirm that you have deposited or provided access to graph files (e.g. BED files) for the called peaks.

Data access links
May remain private before publication.

GEO accession GSE205904:
https://www.ncbi.nlm.nih.gov/geo/query/acc.cgi?acc=GSE205904

Files in database submission

S9CC13_R4_dTAG0h_K27ac_1.fastq.gz
S9CC13_R4_dTAG0h_K27ac_2.fastq.gz
S9CC13_R4_dTAG0h_input_1.fastq.gz
S9CC13_R4_dTAG0h_input_2.fastq.gz
S9CC13_R4_dTAG24h_K27ac_1.fastq.gz
S9CC13_R4_dTAG24h_K27ac_2.fastq.gz
S9CC13_R4_dTAG24h_input_1.fastq.gz
S9CC13_R4_dTAG24h_input_2.fastq.gz
S9CC13_R4_dTAG3h_K27ac_1.fastq.gz
S9CC13_R4_dTAG3h_K27ac_2.fastq.gz
S9CC13_R4_dTAG3h_input_1.fastq.gz
S9CC13_R4_dTAG3h_input_2.fastq.gz
S9CC47_R5_dTAG0h_K27ac_1.fastq.gz
S9CC47_R5_dTAG0h_K27ac_2.fastq.gz
S9CC47_R5_dTAG0h_input_1.fastq.gz
S9CC47_R5_dTAG0h_input_2.fastq.gz
S9CC47_R5_dTAG24h_K27ac_1.fastq.gz
S9CC47_R5_dTAG24h_K27ac_2.fastq.gz
S9CC47_R5_dTAG24h_input_1.fastq.gz
S9CC47_R5_dTAG24h_input_2.fastq.gz
S9CC47_R5_dTAG3h_K27ac_1.fastq.gz
S9CC47_R5_dTAG3h_K27ac_2.fastq.gz
S9CC47_R5_dTAG3h_input_1.fastq.gz
S9CC47_R5_dTAG3h_input_2.fastq.gz
S9CC47_R5_untr_V5.bin10.bw.gz
S9CC47_R5_untr_V5_1.fastq.gz
S9CC47_R5_untr_V5_2.fastq.gz
S9CC47_R5_untr_input_1.fastq.gz
S9CC47_R5_untr_input_2.fastq.gz
WT_R4_dTAG0h_K27ac_1.fastq.gz
WT_R4_dTAG0h_K27ac_2.fastq.gz
WT_R4_dTAG0h_input_1.fastq.gz
WT_R4_dTAG0h_input_2.fastq.gz
WT_R4_dTAG24h_K27ac_1.fastq.gz
WT_R4_dTAG24h_K27ac_2.fastq.gz
WT_R4_dTAG24h_input_1.fastq.gz
WT_R4_dTAG24h_input_2.fastq.gz
WT_R4_dTAG3h_K27ac_1.fastq.gz
WT_R4_dTAG3h_K27ac_2.fastq.gz
WT_R4_dTAG3h_input_1.fastq.gz
WT_R4_dTAG3h_input_2.fastq.gz
WT_R5_dTAG0h_K27ac_1.fastq.gz
WT_R5_dTAG0h_K27ac_2.fastq.gz
WT_R5_dTAG0h_input_1.fastq.gz
WT_R5_dTAG0h_input_2.fastq.gz
WT_R5_dTAG24h_K27ac_1.fastq.gz
WT_R5_dTAG24h_K27ac_2.fastq.gz
WT_R5_dTAG24h_input_1.fastq.gz

WT_R5_dTAG24h_input_2.fastq.gz
WT_R5_dTAG3h_K27ac_1.fastq.gz
WT_R5_dTAG3h_K27ac_2.fastq.gz
WT_R5_dTAG3h_input_1.fastq.gz
WT_R5_dTAG3h_input_2.fastq.gz
WT_R5_untr_V5.bin10.bw.gz
WT_R5_untr_V5_1.fastq.gz
WT_R5_untr_V5_2.fastq.gz
WT_R5_untr_input_1.fastq.gz
WT_R5_untr_input_2.fastq.gz
all.sub.150bpclust.greater2.500bp.merge.k27ac.txt.gz
C13R18_0nM_V5_1.fq.gz
C13R18_0nM_V5_2.fq.gz
C13R18_0nM_in_1.fq.gz
C13R18_0nM_in_2.fq.gz
C13R18_1p6nM_V5_1.fq.gz
C13R18_1p6nM_V5_2.fq.gz
C13R18_1p6nM_in_1.fq.gz
C13R18_1p6nM_in_2.fq.gz
C13R18_500nM_V5_1.fq.gz
C13R18_500nM_V5_2.fq.gz
C13R18_500nM_in_1.fq.gz
C13R18_500nM_in_2.fq.gz
C13R18_5nM_V5_1.fq.gz
C13R18_5nM_V5_2.fq.gz
C13R18_5nM_in_1.fq.gz
C13R18_5nM_in_2.fq.gz
C13R21_0nM_V5_1.fq.gz
C13R21_0nM_V5_2.fq.gz
C13R21_0nM_in_1.fq.gz
C13R21_0nM_in_2.fq.gz
C13R21_1p6nM_V5_1.fq.gz
C13R21_1p6nM_V5_2.fq.gz
C13R21_1p6nM_in_1.fq.gz
C13R21_1p6nM_in_2.fq.gz
C13R21_500nM_V5_1.fq.gz
C13R21_500nM_V5_2.fq.gz
C13R21_500nM_in_1.fq.gz
C13R21_500nM_in_2.fq.gz
C13R21_5nM_V5_1.fq.gz
C13R21_5nM_V5_2.fq.gz
C13R21_5nM_in_1.fq.gz
C13R21_5nM_in_2.fq.gz
S9C13_R18_0nM_AP_1.fq.gz
S9C13_R18_0nM_AP_2.fq.gz
S9C13_R18_0nM_T1_1.fq.gz
S9C13_R18_0nM_T1_2.fq.gz
S9C13_R18_0nM_in_1.fq.gz
S9C13_R18_0nM_in_2.fq.gz
S9C13_R18_1p6nM_AP_1.fq.gz
S9C13_R18_1p6nM_AP_2.fq.gz
S9C13_R18_1p6nM_T1_1.fq.gz
S9C13_R18_1p6nM_T1_2.fq.gz
S9C13_R18_1p6nM_in_1.fq.gz
S9C13_R18_1p6nM_in_2.fq.gz
S9C13_R18_500nM_AP_1.fq.gz
S9C13_R18_500nM_AP_2.fq.gz
S9C13_R18_500nM_T1_1.fq.gz
S9C13_R18_500nM_T1_2.fq.gz
S9C13_R18_500nM_in_1.fq.gz
S9C13_R18_500nM_in_2.fq.gz
S9C13_R18_5nM_AP_1.fq.gz
S9C13_R18_5nM_AP_2.fq.gz
S9C13_R18_5nM_T1_1.fq.gz
S9C13_R18_5nM_T1_2.fq.gz
S9C13_R18_5nM_in_1.fq.gz
S9C13_R18_5nM_in_2.fq.gz
S9C13_R21_0nM_AP_1.fq.gz
S9C13_R21_0nM_AP_2.fq.gz
S9C13_R21_0nM_T1_1.fq.gz
S9C13_R21_0nM_T1_2.fq.gz
S9C13_R21_0nM_in_1.fq.gz
S9C13_R21_0nM_in_2.fq.gz
S9C13_R21_1p6nM_AP_1.fq.gz
S9C13_R21_1p6nM_AP_2.fq.gz
S9C13_R21_1p6nM_T1_1.fq.gz

S9C13_R21_1p6nM_T1_2.fq.gz
S9C13_R21_1p6nM_in_1.fq.gz
S9C13_R21_1p6nM_in_2.fq.gz
S9C13_R21_500nM_AP_1.fq.gz
S9C13_R21_500nM_AP_2.fq.gz
S9C13_R21_500nM_T1_1.fq.gz
S9C13_R21_500nM_T1_2.fq.gz
S9C13_R21_500nM_in_1.fq.gz
S9C13_R21_500nM_in_2.fq.gz
S9C13_R21_5nM_AP_1.fq.gz
S9C13_R21_5nM_AP_2.fq.gz
S9C13_R21_5nM_T1_1.fq.gz
S9C13_R21_5nM_T1_2.fq.gz
S9C13_R21_5nM_in_1.fq.gz
S9C13_R21_5nM_in_2.fq.gz
all.sub.150bpclust.greater2.500bp.merge.SOX9titr.TWIST1.TFAP2A.in.counts.txt.gz
all.sub.150bpclust.greater2.500bp.merge.SOX9titr.V5.counts.txt.gz
S9CC47_R18_0h_V5_1.fq.gz
S9CC47_R18_0h_V5_2.fq.gz
S9CC47_R18_0h_in_1.fq.gz
S9CC47_R18_0h_in_2.fq.gz
S9CC47_R18_3h_V5_1.fq.gz
S9CC47_R18_3h_V5_2.fq.gz
S9CC47_R18_3h_in_1.fq.gz
S9CC47_R18_3h_in_2.fq.gz
S9CC47_R18_0h_V5.bin10.bw
S9CC47_R18_3h_V5.bin10.bw

Genome browser session
(e.g. UCSC)

Provide a link to an anonymized genome browser session for "Initial submission" and "Revised version" documents only, to enable peer review. Write "no longer applicable" for "Final submission" documents.

Methodology

Replicates

Two CNCC differentiation replicates per hESC line

Sequencing depth

Sample #total reads, #uniquely mapped reads, paired-end
S9CC13_R4_dTAG0h_K27ac 19702909 17573660 Paired-end
S9CC13_R4_dTAG0h_input 17171513 14804257 Paired-end
S9CC13_R4_dTAG24h_K27ac 22811174 20455584 Paired-end
S9CC13_R4_dTAG24h_input 4398526 3803373 Paired-end
S9CC13_R4_dTAG3h_K27ac 14035485 12632483 Paired-end
S9CC13_R4_dTAG3h_input 37150935 32120133 Paired-end
S9CC47_R5_dTAG0h_K27ac 16115252 14376813 Paired-end
S9CC47_R5_dTAG0h_input 17131811 14687275 Paired-end
S9CC47_R5_dTAG24h_K27ac 16826112 15029996 Paired-end
S9CC47_R5_dTAG24h_input 16575684 14201610 Paired-end
S9CC47_R5_dTAG3h_K27ac 20208835 17909269 Paired-end
S9CC47_R5_dTAG3h_input 16650520 14398709 Paired-end
WT_R4_dTAG0h_K27ac 23949200 21492733 Paired-end
WT_R4_dTAG0h_input 20173375 17391911 Paired-end
WT_R4_dTAG24h_K27ac 15887196 14220467 Paired-end
WT_R4_dTAG24h_input 16342587 14070696 Paired-end
WT_R4_dTAG3h_K27ac 19413998 17457160 Paired-end
WT_R4_dTAG3h_input 17294971 14924313 Paired-end
WT_R5_dTAG0h_K27ac 16775538 14991098 Paired-end
WT_R5_dTAG0h_input 16300130 14025931 Paired-end
WT_R5_dTAG24h_K27ac 18299418 16349153 Paired-end
WT_R5_dTAG24h_input 18136091 15475742 Paired-end
WT_R5_dTAG3h_K27ac 18473353 16354827 Paired-end
WT_R5_dTAG3h_input 19514872 16736835 Paired-end
S9C13_R18_0nM_AP 5092567 3371626 Paired-end
S9C13_R18_0nM_T1 13220990 10497414 Paired-end
S9C13_R18_0nM_in 10095191 4074233 Paired-end
S9C13_R18_1p6nM_AP 10689895 7529710 Paired-end
S9C13_R18_1p6nM_T1 19014598 15056178 Paired-end
S9C13_R18_1p6nM_in 8996062 3907893 Paired-end
S9C13_R18_500nM_AP 8297763 5490568 Paired-end
S9C13_R18_500nM_T1 12576772 9757678 Paired-end
S9C13_R18_500nM_in 12139794 4744689 Paired-end
S9C13_R18_5nM_AP 3898204 2683774 Paired-end
S9C13_R18_5nM_T1 9432451 7075177 Paired-end
S9C13_R18_5nM_in 11954385 4772522 Paired-end
S9C13_R21_0nM_AP 5621370 3323295 Paired-end
S9C13_R21_0nM_T1 6117521 4051422 Paired-end
S9C13_R21_0nM_in 13494864 5921549 Paired-end

	S9C13_R21_1p6nM_AP 18776354 14814544 Paired-end<br>S9C13_R21_1p6nM_T1 3332709 2023755 Paired-end<br>S9C13_R21_1p6nM_in 10455700 4827845 Paired-end<br>S9C13_R21_500nM_AP 11196045 8037365 Paired-end<br>S9C13_R21_500nM_T1 6325174 4234446 Paired-end<br>S9C13_R21_500nM_in 9677671 4044655 Paired-end<br>S9C13_R21_5nM_AP 12200307 9322397 Paired-end<br>S9C13_R21_5nM_T1 8421010 6139030 Paired-end<br>S9C13_R21_5nM_in 10498564 5010970 Paired-end<br>C13R18_0nM_V5 8596070 4080234 Paired-end<br>C13R18_0nM_in 5190485 1500706 Paired-end<br>C13R18_1p6nM_V5 8302768 4014248 Paired-end<br>C13R18_1p6nM_in 5668285 1664329 Paired-end<br>C13R18_500nM_V5 7220039 3521834 Paired-end<br>C13R18_500nM_in 5122178 1379264 Paired-end<br>C13R18_5nM_V5 7585309 3988019 Paired-end<br>C13R18_5nM_in 4447450 1246852 Paired-end<br>C13R21_0nM_V5 6675128 3692716 Paired-end<br>C13R21_0nM_in 7359899 2048934 Paired-end<br>C13R21_1p6nM_V5 7023127 2870076 Paired-end<br>C13R21_1p6nM_in 5679400 1588508 Paired-end<br>C13R21_500nM_V5 8901195 4456518 Paired-end<br>C13R21_500nM_in 6418679 1712338 Paired-end<br>C13R21_5nM_V5 6984077 3624310 Paired-end<br>C13R21_5nM_in 4773704 1344702 Paired-end
Antibodies	Abcam rabbit anti-V5 (ab9116, ab15828), ActiveMotif anti-H3K27ac (39133), Abcam mouse anti-TWIST1 (ab50887)
Peak calling parameters	For TWIST1 ChIP peaks were called using MACS2 with default parameters; In other cases ChIP counts were calculated over reproducible ATAC-seq peaks as described in methods
Data quality	Effects of SOX9 depletion on H3K27ac ChIP-seq signal were observed to be highly correlated with effects on ATAC-seq signal at the same regulatory elements (Spearman rho 0.74 for 3h depletion, 0.82 for 24h depletion).
Software	skewer v0.2.2; bowtie2 v2.4.1; samtools v1.10; deeptools v3.5.0; MACS2 v2.2.7.1; processed data and code is available on Zenodo (10.5281/zenodo.6596465)

Flow Cytometry

Plots

Confirm that:

☒ The axis labels state the marker and fluorochrome used (e.g. CD4-FITC).

☒ The axis scales are clearly visible. Include numbers along axes only for bottom left plot of group (a 'group' is an analysis of identical markers).

☒ All plots are contour plots with outliers or pseudocolor plots.

☒ A numerical value for number of cells or percentage (with statistics) is provided.

Methodology

Sample preparation	CNCCs were harvested for flow cytometry using accutase and quenching with FACS buffer (5% FBS in PBS). Chondrocytes were harvested as described previously with the following modifications. Chondrocytes were incubated in digestion medium for ~1hr with gentle agitation every 15 min. Digestion medium: DMEM-KO, 1mg/mL Pronase (Roche, 11459643001), 1mg/mL Collagenase B (Roche, 11088815001), 4U/mL Hyalauronidase (Sigma, H3506-500mg). Digested cells were then washed twice in PBS
Instrument	Beckman Coulter Cytoflex V2-B3-R2
Software	Collection: CytExpert Acquisition and Analysis Software v2.4<br>Analysis: FCSalyzer v0.9.18
Cell population abundance	Between 80% (CNCCs) and 50% (chondrocytes) passed the FSC/SSC gating used for analyzing live cells. Cells were not sorted, only analyzed for FITC signal (representing mNeonGreen fluorescence)
Gating strategy	Cells were gated based on FSC and SSC to select viable cells with known size and scatter properties as previously observed in CNCCs and chondrocytes, which was also shown to select viable cells in preliminary studies with 7-AAD viability staining. The FSC/SSC-gated cells were then analyzed for FITC signal (representing mNeonGreen fluorescence)

☒ Tick this box to confirm that a figure exemplifying the gating strategy is provided in the Supplementary Information.
