# Supplementary material for: Precise modulation of transcription factor levels identifies features underlying dosage sensitivity
Source: Nat Genet. 2023 Apr 6;55(5):841–51. doi: 10.1038/s41588-023-01366-2 (PMC10181932; doi:10.1038/s41588-023-01366-2)
Supplement: Source Data Fig. 1 — Unprocessed western blots. [file 41588_2023_1366_MOESM5_ESM.pdf]

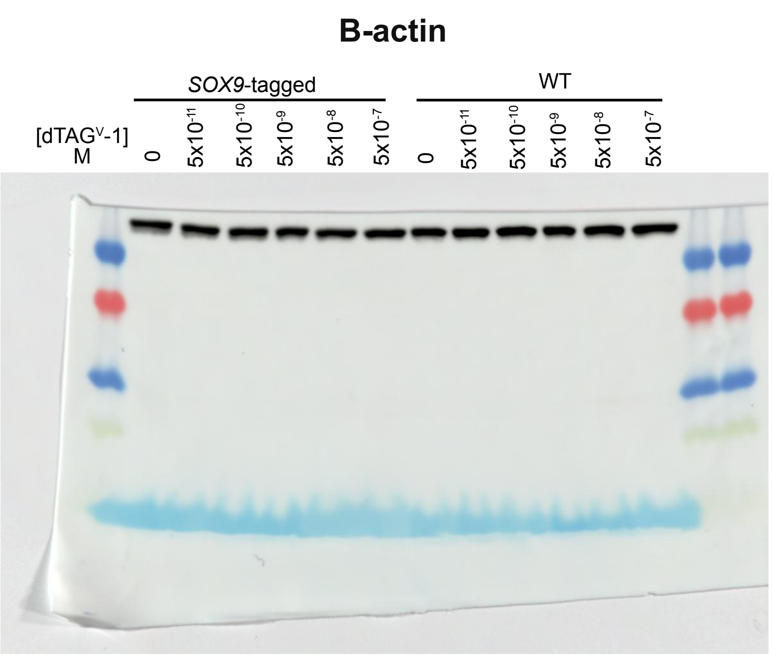
B-actin
SOX9-tagged
WT
[dTAGV-1]
M
0
5x10-11
5x10-10
5x10-9
5x10-8
5x10-7
0
5x10-11
5x10-10
5x10-9
5x10-8
5x10-7

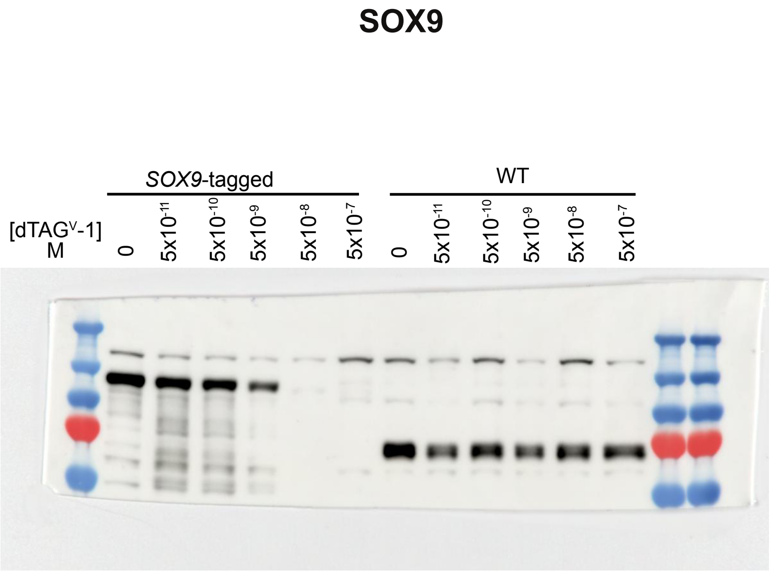
SOX9
SOX9-tagged
WT
[dTAGV-1]
M
0
5x10-11
5x10-10
5x10-9
5x10-8
5x10-7
0
5x10-11
5x10-10
5x10-9
5x10-8
5x10-7
