# Supplementary figures and images for: Precise modulation of transcription factor levels identifies features underlying dosage sensitivity
Source: Nat Genet. 2023 Apr 6;55(5):841–51. doi: 10.1038/s41588-023-01366-2 (PMC10181932; doi:10.1038/s41588-023-01366-2)

Clones 1-24

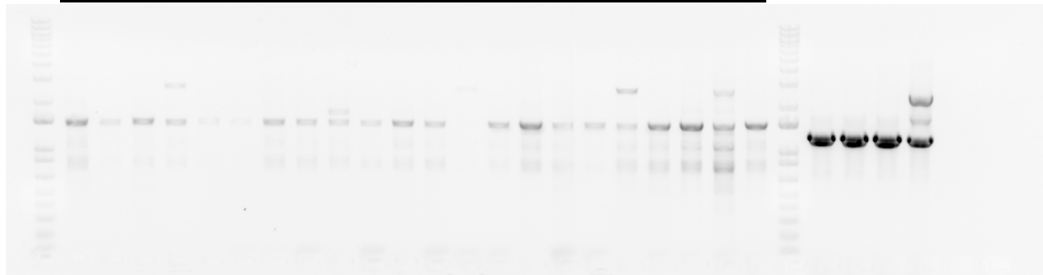

Clones 25-48

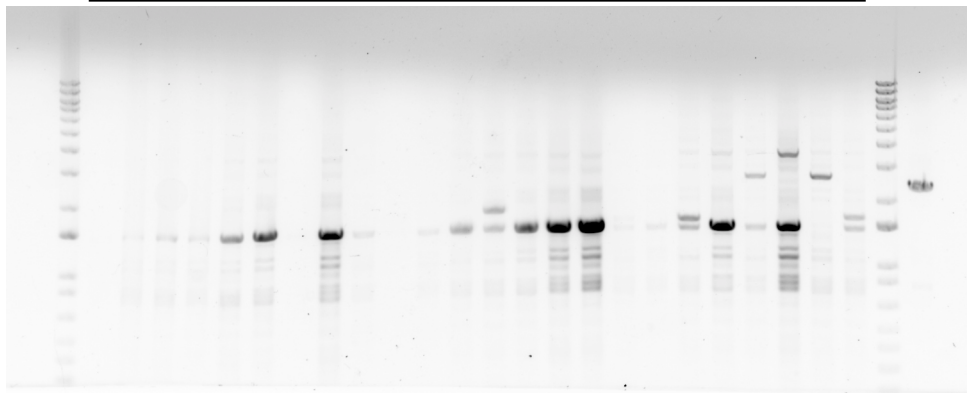

Supplement: Source Data Extended Data Fig. 1 — Unprocessed gels. [file 41588_2023_1366_MOESM6_ESM.pdf]
